# The Estimation of Phenotypic and Genetic Parameters for Growth Rate Traits of Abergelle Goat Under the Community‐Based Breeding Programme in the North‐Eastern Parts of Ethiopia

**DOI:** 10.1002/vms3.71202

**Published:** 2026-08-29

**Authors:** Yeshiwas Walle Abebe, Wossenie Shibabaw Meberatie, Zeleke Tesema Wondie

**Affiliations:** ^1^ Bahir Dar University College of Agriculture and Environmental Sciences Bahir Dar Ethiopia; ^2^ Amhara Agricultural Research Institute Sekota Dry Land Agricultural Research Center Sekota Ethiopia; ^3^ Amhara Agricultural Research Institute Debere Birhan Agricultural Research Center Debere Berhan Ethiopia

**Keywords:** body weight, genetic correlation, genetic parameter, growth rate, heritability

## Abstract

**Background:**

Understanding phenotypic performance and genetic parameter estimation is important for effective genetic progress in animal breeding and genetics. The data were collected for 6 years (2018–2023) from the established village of Abergelle goat community‐based breeding programme (CBBP) area in Sekota Dryland Agricultural Research Center. The investigated traits were birth weight (BWT), 3‐month weight (TMWT), 6‐month weight (SMWT), 9‐month weight (NMWT), yearling weight (YWT), average daily weight gain from birth to 3 months (ADG1), average daily weight gain from 3 to 6 months (ADG2), average daily weight gain from 6 to 9 months (ADG3) and average daily weight gain from 9 months up to yearling age (ADG4).

**Objectives:**

To evaluate the phenotypic performance and genetic parameter estimation of growth rate traits of Abergelle goat breeds in CBBP in scale‐up villages in north‐eastern parts of Ethiopia.

**Methods:**

Four univariate animal models were fitted using the average information restricted maximum likelihood (AI‐REML) procedure to estimate variance components.

**Results:**

The overall least square mean (LSM) ± standard error (SE) of ADG1, ADG2, ADG3 and ADG4 were 58.2 ± 2.27, 37.8 ± 2.04, 19.2 ± 0.7 and 10.5 ± 0.12 g per day, respectively. The direct heritability estimates for BWT, TMWT, SMWT, NMWT, YWT, ADG1, ADG2, ADG3 and ADG4 were 0.34 ± 0.070, 0.45 ± 0.062, 0.40 ± 0.040, 0.42 ± 0.07, 0.41 ± 0.14, 0.18 ± 0.04, 0.03 ± 0.001, 0.19 ± 0.04 and 0.19 ± 0.04, respectively. Genetic correlations among BWT, TMWT, SMWT, NMWT and YWT were in the range of 0.068–0.84 and phenotypic correlations were also in the range of 0.23–0.89. Genetic correlations for BWT was moderate and higher among SMWT, NMWT and YWT, indicating correlated responses. Thus, BWT had no strong link to later growth in Abergelle goats under CBBP scale‐up village.

**Conclusion:**

The moderate to high direct heritability estimates indicate that selection for growth would be effective in achieving genetic improvement.

## Graphical abstract

Genetic and phenotypic parameters for growth traits of Abergelle goats were estimated using 6 years of data from community‐based breeding programme in scale‐up villages. Growth traits showed positive genetic and phenotypic correlations, with moderate to high heritability, indicating strong potential for genetic improvement through selection. However, birth weight had weak associations with later growth traits, suggesting it is a limited predictor of subsequent performance.

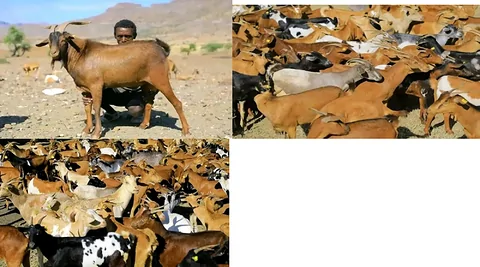

## 1 Introduction

Goats thrive across the broadest ecological zones. They can survive and reproduce in remote and marginal lands often unsuitable for crop farming. Goats play an important role in smallholder production systems because they require relatively low initial investment, can efficiently utilize locally available feed resources, and provide affordable sources of food and income through the production of meat, milk, and fiber. They also have relatively high growth rate, reach market age quickly and are easy for most family members to manage (Mohammed et al. 2013). Tropical Africa is largely characterized by extensive production system and typically low input–output system, which mainly depends on indigenous goat genetic resources (Sheriff et al. 2020; Philipsson et al. 2011).

Ethiopia's large goat flock size was estimated to be 52.4 million (CSA 2021). They have been found in all agro‐ecological zones. About one herd of goats in Ethiopia are found in the highlands. Almost all goat population is managed by resource‐poor smallholder farmers and pastoralists under traditional and extensive production system. In addition, smallholder farmers keep small ruminants for cash income, as living bank against failure of crop production and for meat and milk consumption for their families (Abegaz et al. 2014). But meat production per animal in Ethiopia was 9 kg which is less than Kenya and Sudan by 3 and 6 kg, respectively. The total annual meat production from small ruminants was 154,000 tonnes per year (Rekik et al. 2016). It is relatively small compared with total flock size. The annual milk production from goats in Ethiopia is 93,000 metric tonnes which is more than Kenya (91,000 metric tonnes) and less than Sudan (154,400 metric tonnes) (Banerjee et al. 2000). It was suggested that the difference in production of milk and meat is due to genetic potential, feed availability, nutrition and the overall farming practice the producers followed. Therefore, it is very important to look for options that increase the economical contribution of goats to farmers through performance evaluation of the animals (Alemayehu et al. 2021).

Abergelle goats are ever‐present and an important component to the subsistence and economic and social livelihoods of the poor rural farmers in Wag‐Himera zone. This breed has been found in all agro‐ecologies and farming systems of Wag‐Himera, along with Tekeze river, Gondar, east Belsa and in some parts of Tigray region (Taye et al. 2013). In all parts of the study area, goats are raised as a major source of cash income, milk, meat and manure for crop production (Abegaz et al. 2014). In Wag‐Himera zone, the goat population was estimated to be above 500,000 (CSA 2021). Larger goat flocks were reported in the lowland areas of Wag‐Himra, with an average of 27 goats per household (Abegaz et al. 2014). The breed is characterized by its small body size and compacted body structure and high adaptation to the harsh environment, and production of meat and milk for their producers in addition to cash income. Abergelle goat breed exhibit relatively low reproductive performance, with an average litter size close to 1.06–1.11 kids per kidding and predominance of single births (Belay et al. 2014; Deribe and Taye 2014). The average kidding interval for Abergelle goats is about 11.3 months, indicating that it typically produces one kid per year (Deribe and Taye 2014). Twinning ability in this breed is limited, with only about 10.24% of births resulting in twins, reflecting a low twinning rate compared to other Ethiopian goat breeds (Abegaz et al. 2014).

Currently, the demand of red meat from small ruminants especially goat ‘*kurete’* houses and hotels are increasing in domestic markets and Abergelle goat is contributing much more red meat supply to the local market. In 2022, Ethiopia produced a total of 747,544 tonnes of meat, of which most (698,189 tonnes) came from red meat animals. Cattle accounted for 419,868 tonnes, sheep and goats for 242,774 tonnes and camels for 35,545 tonnes. Overall, these red meat animals contributed 93% of the country's total meat production (MOA 2024). In the study area, selective breeding was implemented according to the breeding objective of farmers (Abegaz et al. 2014; Tesema et al. 2024). Scaling up community‐based breeding programmes (CBBP) in participatory manner is the best approach for sustainable breed improvement programmes in the tropics (Mueller et al. 2019; Kaumbata et al. 2020).

The potential for genetic improvement in economically important traits of goats through selection depends on the extent of the genetic variation and estimates of the genetic and phenotypic parameters in goat breeding (Kebede et al. 2012). However, before selection, knowledge of genetic variation and genetic parameters are helpful in determining the method of selection, to predict direct and correlated response to selection, choosing a breeding system to be adopted for future improvement as well as in the estimation of genetic gain (Tesema et al. 2020). Therefore, the objective of this research study was to estimate genetic and phenotypic parameters for growth and milk production traits of Abergelle goats in CBBP in scale‐up villages.

## 2 Materials and Methods

### 2.1 Description of the Study Area

The study was conducted on farms by Sekota Dry Land Agricultural Research Center (SDARC) in Addis Mender and Alquzu villages in Sekota and Ziquala districts, respectively. Sekota and Ziquala districts were found in Wag‐Himera zone in Amhara region in the north‐eastern parts of Ethiopia. Sekota district is located 720 km away from Addis Ababa and 430 km from Bahir Dar capital city of the Amhara Region at an altitude of 2200 m.a.s.l and at 12°41′11.92″ N and 39°00′58″ E. Annual rainfall is 1131 mm per year. The pattern and distribution of the rainfall is erratic and uneven. Average temperature ranges from 9.5°C to 33.55°C. The vegetation can be characterized as being semi‐arid shrubs dominated by various Acacia species with a sparse ground cover of annual grasses. The district is characterized by long dry season lasting from December to June (Taye et al. 2013).

Ziquala district is located at 12°48′41.39″ N and 38°43′22.02″ E 775 km north‐east of Addis Ababa, Ethiopia. The district has rugged topography characterized by mountains, steep escarpments and deeply incised valleys. Mixed crop‐livestock production system with high priority of livestock production is the major farming practice in the area. The mean annual rainfall of the area was 250—650 mm with very short and erratic distribution from early July to late August. The mean annual temperature is 25°C—39°C and an altitude of 1308 m.a.s.l. Based on the above agro‐meteorological data and feed availability, the area was considered wet from late July to November and the remaining months as dry season (Walle et al. 2024).

In Sekota and Ziquala districts, in the respective villages of Addis Mender and Alquzu, scaling up of CBBP was established in 2018 on Abergelle goat breed. Recording of performance data and selection of breeding bucks were implemented for 6 consecutive years (2018–2023). During selection of breeding bucks, participation of farmers and interest, the economic weight given to their animals in terms of trait when selecting breeding animals’ growth, milk yield, colour and morphological features of the animals were considered. Breeding objective and trait preference of farmers was studied by Abegaz et al. (2014). The established scale‐up CBBP villages which are Addis Mender and Alquzu had 40 and 36 farmers involved in each village, respectively.

### 2.2 Animal Management

The goat flocks examined in this study were typically managed by members of farmers in the scale‐up CBBP villages. Animals primarily sourced their feed from browsing and grazing on natural pasture, shrubs and cactus trees, as well as from residues of legume crops like cowpea and sorghum stover. Improved forage and grass were occasionally provided in small quantities by breeder farmers. The location of the flock's housing varied depending on the season and was constructed from locally available materials, typically situated at a distance from the farmers' residences and some farmers used caves as housing for their animals.

Participants in the scale‐up CBBP village received free veterinary services from partner non‐governmental organizations like ICARDA. Goats underwent deworming for internal parasites twice a year, in January and June. External parasite control measures were administered as needed when infestations occurred. Annual vaccinations were provided against diseases such as blackleg, contagious caprine pleuropneumonia and foot and mouse disease, while vaccinations for pasteurellosis, pneumonia and peste des petits in ruminants were given biannually. Broad‐spectrum antibiotics (oxytetracycline L.A.) were administered before and after the rainy seasons. Multivitamin injections were given whenever signs of stress were detected. Health professionals along with participant farmers, data collectors and enumerators monitored the health status of the animals and dealt with the symptoms of diseases for veterinarians.

Breeding bucks were selected annually on a predetermined date based on performance data recorded by enumerators. The selection process focused on overall excellence of animal phenotype, colour, appearance, highest estimated breeding value (EBV) and sexual behaviour (Abegaz et al. 2014). Since 2020, selection has been based on EBVs of candidate breeding animals. Two‐step selections were used to select improved bucks for breeding as a sire. Initially, candidate bucks were pre‐selected and ranked based on their 6‐month weight (SMWT). Subsequently, they were re‐evaluated and ranked based on their yearling weight (YWT) EBVs. The top 10% of superior bucks were retained for breeding, while others were either sold for breeding purposes or castrated to prevent unwanted mating. Selected breeding bucks typically served for 4–5 years with 1‐year service in the same flock, and agreements were signed between buck holders and village CBBP members outlining their responsibilities. After a year of service, breeding bucks were redistributed to other farmers to mitigate the effects of inbreeding within the community or village CBBP members.

### 2.3 Data Source and Experimental Design

**FIGURE 1:**
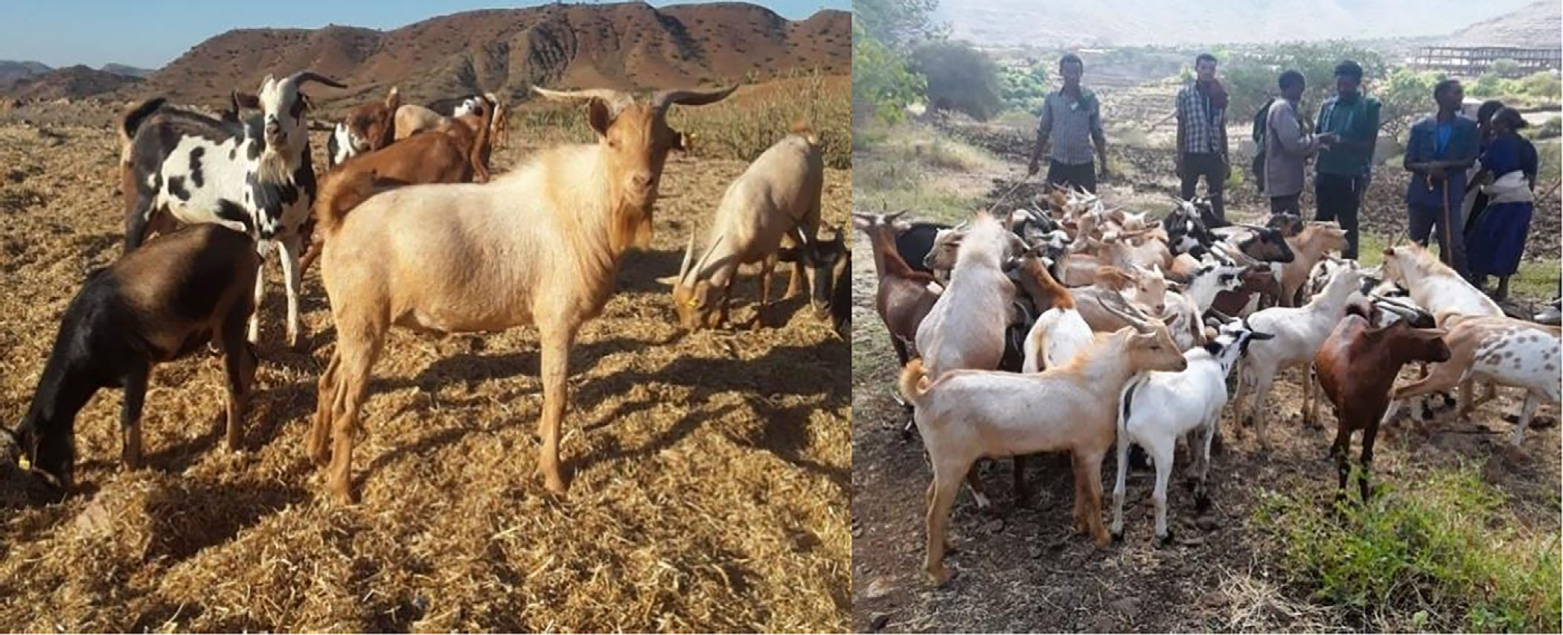
During buck selection (right) and after mating season (left) in Addis Mender village.

**FIGURE 2:**
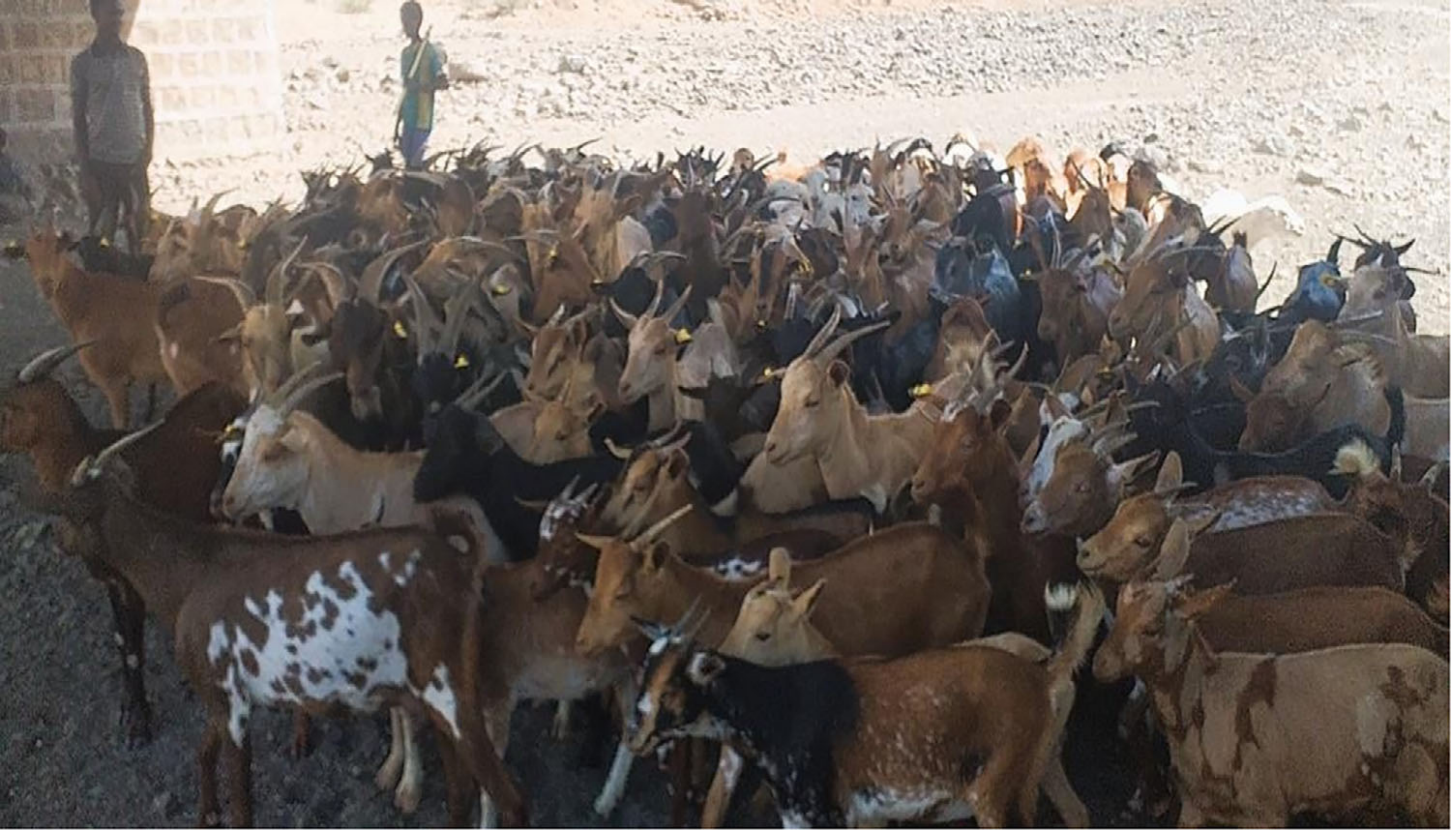
During mating season in Alquzu village in one‐member farmer's flocks.

The whole performance and pedigree data for the study were obtained from this ongoing scale‐up CBBP village in Addis Mender and Alquzu in Sekota and Ziquala districts, respectively, under SDARC. The research staff of the programme from SDARC and other partner institutes like ICARDA were carrying regular follow‐up of both functionality of scale‐up villages and record‐keeping practices by enumerators. Performance data were recorded, as per performance record format prepared by ICARDA. The performance data along with pedigree information are being maintained in the data recording book of the individual village. The data routinely collected by the enumerators were the relevant information about newborn kids such as owners at birth (which are the names of farmers), birth date, kid sex, birth type, kids’ birth weight (BWT), doe parity, sires ID and doe ID. Periodically collected data were checked before being entered into the computer and reported to SDARC at least two or three times in a month. The data utilized in the present study were (i) growth trait data, namely, BWT, 3‐month weight (TMWT), SMWT, 9‐month weight (NMWT), YWT, daily milk yield, 90‐days total milk yield and lactation length. The selection of breeding bucks after mating and during the mating season is illustrated in Figures 1 and 2, respectively.

BWT of kids was recorded within 24 h of birth. To standardize the data, all growth traits (TMWT, SMWT, NMWT and YWT) were adjusted to fixed ages of 90, 180, 270 and 360 days, respectively. Pedigree information was assessed for reliability and consistency using Pedigree Viewer software (Kinghorn and Kinghorn 2015), with records containing duplicates or bisexuality removed. Parity was categorized as 1 to ≥ 7, with observations of parity above 7 grouped together as parity ≥ 7 due to limited sample size. Similarly, birth type was classified as 1 and 2. The fixed effect of kidding/birth season was classified into two classes—wet and dry (Deribe and Taye 2014). The seasons were categorized into dry (beginning of January–end of June) and wet (beginning of July–end of December) based on the availability of browse, crop aftermath and rainfall amount.

### 2.4 Data Adjustment

Animals are rarely weighed on exactly the same day; if one kid is weighed at 95 days and another at 110 days, their raw weights are not directly comparable. Adjusting both to 90 days gives an estimate of what each would weigh at the same age, which helps compare growth rate rather than just age at weighing. Standard‐age adjustment is a common approach in livestock performance recording and genetic evaluations. TMWT, SMWT, NMWT and YWT were adjusted at fixed ages of 90, 180, 270 and 360 days, respectively, as follows:

$$\mathrm{Adjusted}\;3\;\mathrm{month}\;\mathrm{weight}\;\left(\mathrm{kg}\right)=\frac{90\left(\mathrm{TMWT}\;-\;\mathrm{BWT}\right)}{\;D+\;\mathrm{BWT}}$$

$$\mathrm{Adjusted}\;6\;\mathrm{month}\;\mathrm{weight}\;\left(\mathrm{kg}\right)=\frac{180\left(\mathrm{SMWT}\;-\;\mathrm{BWT}\right)}{\;D+\;\mathrm{BWT}}$$

$$\mathrm{Adjusted}\;9\;\mathrm{month}\;\mathrm{weight}\;\left(\mathrm{kg}\right)=\frac{270\left(\mathrm{NMWT}\;-\;\mathrm{BWT}\right)}{\;D+\;\mathrm{BWT}}$$

$$\mathrm{Adjusted}\;\mathrm{yearling}\;\mathrm{weight}\;\left(\mathrm{kg}\right)=\frac{\;360\left(\mathrm{YWT}\;-\;\mathrm{BWT}\right)}{\;D+\;\mathrm{BWT}}$$

where BWT is birth weight, TMWT is 3‐month weight on given fixed date, SMWT is 6‐month weight on given fixed date, NMWT is 9‐month weight on fixed date, YWT is yearling weight on fixed date and *D* is the number of days between weighing date and date of birth.

The average daily body weight gain from birth to 3 months of age, 3 to 6 months of age, 6 to 9 months of age and 9 months to YWT age has been estimated as follows:

(1) $$\mathrm{ADG}1\;\left(\frac{g}{\mathrm{day}}\right)=\frac{\mathrm{TMWT}\;-\;\mathrm{BWT}}{90}\;\;\times \;1000$$

Average daily weight gain TMWT up to SMWT age:

(2) $$\mathrm{ADG}2\;\left(\frac{g}{\mathrm{day}}\right)=\frac{\mathrm{SMWT}\;-\;\mathrm{TMWT}}{90}\;\times \;1000$$

Average daily weight gain SMWT up to NMWT age:

(3) $$\mathrm{ADG}3\;\left(\frac{g}{\mathrm{day}}\right)=\frac{\;\mathrm{NMWT}\;-\;\mathrm{SMWT}}{90}\;\times \;1000$$

Average daily weight gain NMWT up to YWT age:

(4) $$\mathrm{ADG}4\;\left(\frac{g}{\mathrm{day}}\right)=\frac{\mathrm{YWT}\;-\;\mathrm{NMWT}}{90}\;\times \;1000$$

### 2.5 Statistical Analysis of Data

#### 2.5.1 Effects of Non‐Genetic Factors

Data used for the analysis included BWT, TMWT, SMWT, NMWT and YWT. Before conducting the main analysis, data were coded and entered into the computer for analysis and preliminary data analysis; normality test was employed. Then, data were analyzed using the general linear model (GLM) procedure of SAS version 9.0. The non‐genetic factors used in the model included year of birth/kidding (2018–2023), season (wet and dry season), sex (male and female), parity (1, 2, 3, 4, 5, 6 and ≥ 7), birth type (single and twin) and sites (Alquzu and Addis Mender). The Tukey test was used to separate least squares means (LSM). Thus, the following statistical model was used.

Model for growth and weight gain traits:

$$Y_{ijklmn}=\mu +P_{i}+S_{j}+B_{k}+Yr_{l}+S_{em}+S_{xn}+e_{ijklmn}$$

where *Y*_ijklmn_ is the growth and daily weight gain traits for each animal, *μ* is the overall mean, *P_i_* is the *i*th parity (*i* = 7; 1, 2, 3, 4, 5, 6 and ≥ 7), *S_j_* is the *j*th l sites (*j* = Alquzu and Addis Mender), *B_k_* is the *k*th birth type (*k* = 2; single, twin), *Yr_l_* is the *l*th year (*l* = 6; 2018–2023), *S_em_* is the *m*th season (*m* = wet and dry season), *S_xn_* is the *n*th sex (*n* = male and female) and *e*_ijklmn_ is random error.

Description of pedigree structure and performance data used for genetic parameter analyses are presented in Table 1. The parents recorded in the pedigree data include bucks, dams, bucks with its progeny records and dams with its progeny records.

**TABLE 1:** Description of data set for growth traits.

	Traits								
Parameters	BWT	TMWT	SMWT	NMWT	YWT	ADG1	ADG2	ADG3	ADG4
Number of animals	1989	1335	1211	986	846	1335	983	937	700
No of animals with unknown dams	248	248	248	248	248	248	248	248	248
No of animals with unknown sire	251	251	251	251	251	251	251	251	251
Number of sire	54	54	54	48	43	54	54	48	43
Number of dams	635	607	428	404	344	607	428	404	344
Maximum (kg)	3.4	9.6	19.9	24.6	28.4	76	64	40	32
Minimum (kg)	1.1	3.8	7.0	8.6	10.4	48	32	20	12
Mean (kg)	2.06	7.0	7.2	11.5	15.3	62	48	30	22
SD	0.34	0.67	2.12	1.80	3.6	33.4	23.4	24.5	3.6
CV (%)	19.5	18.0	17	19.4	21	27.8	56.4	48.7	40.3

Abbreviations: ADG1, average daily gain from birth to weaning; ADG2, average daily gain from weaning to 6 months; ADG3, average daily gain from 6 to 9 months; ADG4, average daily gain from 9 months to yearling; BWT, birth weight; NMWT, 9‐month weight; SMWT, 6‐month weight; TMWT, 3‐month weight; YWT, yearling weight.

#### 2.5.2 Genetic Parameter Estimation

The variance components and genetic parameters were estimated on model fitting effects of parity, year of birth/year of kidding, season of birth/season of kidding and type of birth, as fixed factors for growth and growth rate traits. Genetic parameters estimation for those traits were estimated using Wombat software (Meyer 2000). Then the fixed effects were fitted in the subsequent models for estimating genetic parameters. Mixed univariate animal models were used to estimate the genetic parameters. Direct additive and maternal additive genetic effects with or without a covariance between them and maternal permanent environmental effects were tested for all traits in different combinations to yield four models. The four models were as follows:

(Model 1) $$y=Xb+Z_{1}a+e$$

(Model 2) $$y=Xb+Z_{1}a+Z_{2}m+ewithCov\left(a,m\right)=0$$

(Model 3) $$y=Xb+Z_{1}a+Z_{2}m+ewithCov\left(a,m\right)=A\sigma_{am}$$

(Model 4) $$y=Xb+Z_{1}a+Z_{2}c+e$$

where *y* is the vector of observed traits of animals; *b*, *a*, *m* and *c* are the vectors of fixed effects, direct additive genetic effects, maternal additive genetic effects and maternal permanent environmental effects, respectively; *X*, *Z*_1_, *Z*_2_ and *Z*3 are the incidence matrices, respectively, relating fixed effects, direct additive genetic effects, maternal additive genetic effects and maternal permanent environmental effects; and *e* is the vector of residuals.

The (co)variance structure for the model was

$$Var\left(a\right)=A\sigma^{2}a,\;Var\left(m\right)=A\sigma^{2}m,\;Var\left(c\right)=IDc^{2}\;\mathrm{and}\;Var\left(\varepsilon \right)=Ike^{2}$$

where *A* is the numerator relationship matrix between animals and ID and IK are identity matrices with orders equal to the number of does and the number of kids, respectively.

Cov (*a*, *m*) indicate whether covariance between direct additive and maternal genetic effects was considered. It was assumed that Var(*a*) = *Aσ* ^2^ *a*; Var(*m*) = *Aσ* ^2^ *m*; Var(*c*) = *Iσ* ^2^ *c*; Var(*e*) = *Iσ* ^2^ *e*, where *I* is the identity matrix, *σ* ^2^ *a* is the direct additive genetic variance, *σ* ^2^ *m* is the maternal additive genetic variance, *σ* ^2^ *c* is the maternal permanent environmental variance and *σ* ^2^ *e* is the residual variance. All components, with the phenotypic variance (*σ* ^2^ *p*) being the sum of *σ* ^2^ *a*, *σ* ^2^ *m*, *σ_am_*, *σ* ^2^ *c* and *σ* ^2^ *e*, were derived at convergence.

Depending on the model, heritability was computed:

$$\mathrm{Direct}\;\mathrm{heritability}\;\mathrm{as}\;h^{2}=\frac{\sigma 2a}{vp\;}$$

$$\mathrm{Maternal}\;\mathrm{heritability}\;m^{2}=\frac{\sigma 2m}{vp\;}$$

The direct‐maternal covariance as proportion of phenotypic variance is *c_am_* = *σ_am_* / *σ* ^2^ *p*. The maternal environmental variance ratio was estimated by the maternal permanent environmental variance as a proportion of *σ* ^2^ *c* (*c* ^2^ = *σ* ^2^ *c* / *σ* ^2^ *p*). The genetic correlation between direct and maternal genetic effects (*r_am_*) is estimated as a ratio of the estimates of the *σam* to the product of the square roots of the estimates of *σ* ^2^ *a* and *σ* ^2^ *m*:

$ram=\frac{\sigma am}{\sqrt{\sigma 2a\;\times \;\sigma 2m}}$ according to Meyer (2000).

Total heritability (*h* ^2^t) was calculated according to the following equation:

$$\;h_{t}^{2}=\frac{\sigma 2a+0.5\;\sigma 2m+1.5\sigma \mathrm{am}}{\sigma 2p}$$

Identifying the optimal model involved employing the likelihood ratio test (LRT). Model comparison based on log likelihood (logL) proved influential when its inclusion significantly enhanced logL compared to neglecting. The logL ratio formula, calculated as twice the maximum likelihood for the full model minus that of the reduced model, alongside chi‐square (*χ* ^2^) with degrees of freedom equal to the difference between the two models, was utilized (Thompson 2008). If logL did not significantly differ (*p* > 0.05), the model with fewer parameters was selected as optimal.

## 3 Results and Discussion

### 3.1 Growth Performance of Abergelle Goat in Scale‐Up CBBP Village

Season, year of birth, parity and sex had significant effects (*p* < 0.001) on BWT (Table 2). Nevertheless, village did not have a significant effect on BWT (*p* > 0.05), but birth type had significant influence (*p* < 0.05) on BWT. Regarding TMWT, SMWT, NMWT and YWT, both village and season demonstrated significant effects (*p* < 0.01). Parity had a significant effect (*p* < 0.05) on TMWT and NMWT but did not have a significant effect on SMWT and YWT. Notably, the type of birth significantly influenced TMWT, while year of birth and sex did not exhibit significant effects on TMWT. Moreover, the birth year and season had highly significant effects (*p* < 0.001) on SMWT, whereas birth type significantly affected TMWT but not SMWT, NMWT and YWT (*p* > 0.05), as indicated in Table 2.

**TABLE 2:** Least squares mean (LSM ± SE) of body weights at different ages of Abergelle goats under up‐scaled CBBPs.

Source of variation	*N*	BWT LSM ± SE	*N*	TMWT LSM ± SE	*N*	SMWT LSM ± SE	*N*	NMWT LSM ± SE	*N*	YWT LSM ± SE
Overall	1989	2.3 ± 0.04	1335	7.5 ± 0.03	1211	8.8 ± 0.040	986	12.1 ± 0.067	846	15.3 ± 0.092
**CV (%)**		16.5		18.08		19.4		17.3		15.6
**Village**		**NS**		***		***		***		***
Addis Mender	793	2.2 ± 0.05	275	8.1 ± 0.038^a^	304	9.8 ± 0.038^a^	230	14.1 ± 0.06^a^	230	16.1 ± 0.17^a^
Alquzu	1145	2.2 ± 0.08	1060	7.3 ± 0.09^b^	907	8.5 ± 0.090^b^	756	11.4 ± 0.12^b^	616	14.9 ± 0.10^b^
**Sex**		***		*		NS		*		*
Male	974	2.3 ± 0.05^a^	671	7.60 ± 0.05^a^	613	9.1 ± 0.12	461	12.8 ± 0.11^a^	461	15.6 ± 0.14^a^
Female	964	2.2 ± 0.07^b^	664	7.47 ± 0.09^b^	598	9 ± 0.13	525	12.6 ± 0.07^b^	525	15.4 ± 0.18^b^
**Type B**		*		*		**NS**		**NS**		**NS**
Single	1785	2.30 ± 0.05^a^	1227	7.8 ± 0.04^a^	1129	9.2 ± 0.05	938	12.7 ± 0.1	812	15.5 ± 0.14
Twin	153	2.20 ± 0.05^b^	108	7.5 ± 0.05^b^	82	9.18 ± 0.05	48	12.6 ± 0.07	34	15.3 ± 0.15
**Season**		*		***		***		*		**
Wet	1287	2.3 ± 0.08^a^	904	8.0 ± 0.04^a^	807	9.3 ± 0.6^a^	652	13.2 ± 0.08^a^	543	15.8 ± 0.103^b^
Dry	651	2.2 ± 0.05^b^	431	7.6 ± 0.04^b^	404	8.9 ± 0.04	334	12.3 ± 0.08^b^	303	14.6 ± 0.16^c^
**Year**		***		*		*		*		*
2018	527	2.2 ± 0.08^c^	319	7.7 ± 0.08^b^	272	8.9 ± 0.08^c^	162	12.8 ± 0.19^a^	117	16 ± 0.24^a^
2019	300	2.3 ± 0.01^b^	208	7.9 ± 0.06^a^	187	9.1 ± 0.09^c^	157	12.6 ± 0.14^b^	144	14.7 ± 0.22^c^
2020	338	2.2 ± 0.01^c^	229	7.9 ± 0.09^a^	212	9.09 ± 0.09^c^	185	12.9 ± 0.14^a^	147	15 ± 0.24^b^
2021	365	2.4 ± 0.01^a^	228	7.9 ± 0.09^a^	218	9.3 ± 0.10^b^	178	12.5 ± 0.16^b^	135	15.4 ± 0.21^b^
2022	408	2.3 ± 0.07^b^	351	7.7 ± 0.06^b^	322	9.2 ± 0.07^b^	304	12.5 ± 0.11^b^	303	15.2 ± 0.141^b^
2023	472	2.1 ± 0.02^d^	384	7.2 ± 0.05^c^	334	9.7 ± 0.04^a^	124	12 ± 0.01^d^	62	15.2 ± 0.13^b^
**Parity**		***		*		**NS**		*		*
1	323	2.2 ± 0.01^c^	239	7.5 ± 0.09^e^	221	8.8 ± 0.08	189	11.8 ± 0.15^c^	158	15.2 ± 0.21^c^
2	330	2.2 ± 0.01^b^	239	7.6 ± 0.06^c^	223	8.8 ± 0.09	175	12.3 ± 0.18^c^	155	15.5 ± 0.24^b^
3	402	2.4 ± 0.04^a^	220	7.7 ± 0.08^b^	214	8.8 ± 0.07	179	12.5 ± 0.15^b^	154	15.2 ± 0.21^c^
4	354	2.3 ± 0.08^a^	242	7.4 ± 0.08^e^	232	8.8 ± 0.10	186	12.2 ± 0.12^b^	168	15.0 ± 0.17^c^
5	229	2.2 ± 0.01^c^	179	7.4 ± 0.09^e^	149	8.9 ± 0.10	126	11.6 ± 0.17^c^	110	15.5 ± 0.27^b^
6	246	2.2 ± 0.01^c^	191	7.6 ± 0.11^d^	148	8.7 ± 0.12	113	11.9 ± 0.19^c^	83	15.1 ± 0.30^b^
≥ 7	54	2.2 ± 0.03^c^	25	8.5 ± 0.34^a^	24	9.2 ± 0.34	18	14.6 ± 0.44^a^	18	16.9 ± 0.62^a^

*Note*: Least squares means with different superscripts within the same column and class are statistically different.

Abbreviations: BWT, birth weight; CV, coefficient of variation; LSM, least squares means; NMWT, 9‐month weight; SMWT, 6‐month weight; TMWT, 3‐month weight; Type B, type of birth; YWT, yearling weight.

NS, *p* > 0.05.

* *p* < 0.05.

** *p* < 0.01.

*** *p* < 0.001.

#### 3.1.1 BWT

Sex, year and parity had a highly significant effect (*p* < 0.001) on BWT. Season and birth type also had a significant effect (*p* < 0.005) on BWT but village had no significant effect (*p* > 0.05). There was an incremental trend on BWT based on type of birth, that is, single‐born kids showed higher BWT than twins. This study was in line with Amare et al. (2020) who reported this for the same breed, and for rift valley family reported by Gatew et al. (2019). According to the authors, the possible reason for these trends should be the limited uterine space during pregnancy with twins than single kid and competition for nutrients in dams especially during the last trimester of pregnancy. In this study, males were heavier than female kids; this should be due to the effect of hormones in males and females. This result is in line with the Central Highland goat breed (Abegaz et al. 2020). The present study revealed that BWT across the years had statistically significant increasing and decreasing ways (*p* < 0.05).

The pair‐wise comparison of BWT among seven parities showed that all differences among all parties were non‐significant except 1–2, 2–4, 2–5, 3–6 and 3–7 parities which had significant difference on BWT. The BWT of Abergelle goat (2.3 ± 0.02 kg) was not comparable with BWT (2.68 ± 0.13 kg) of Central Highland goats (Zergaw et al. 2016) and was greater in BWT than the same family Woyto Guji breed (2.03 kg) (Zergaw et al. 2016). BWT of Abergelle goat in scale‐up CBBP village was found to be greater than the one reported by Deribe and Taye (2013a) for the same breed, which is 2.01 ± 0.03 kg and less than that of Central Highland breed reported by Abegaz et al. (2020), which is 2.66 ± 0.03 kg.

#### 3.1.2 TMWT

Season and village had a highly significant effect on TMWT (*p* < 0.001). Parity, sex and birth type had also showed a significant effect (*p* < 0.05), but years of birth had no significant effect on TMWT, as shown in Table 2. The LSM ± standard error (SE) of TMWT in two scale‐up villages were 8.13 ± 0.038 and 7.38 ± 0.09 kg in Addis Mender and Alquzu village, respectively. The comparison of the means showed that the TMWT difference between villages were highly significant; this may be due to variation in management and availability of feed in the study village. Single‐born kids were 7.85 ± 0.053 kg, which is heavier than twin‐born ones (7.51 ± 0.05 kg). This effect may be attributed to lesser availability of uterine space for twin births, prenatal nutrition/development and also competition for dams' milk during pre‐weaning period (Tesema et al. 2021). Male kids were insignificantly heavier than female kids in TMWT, both male and female kids experienced shock due to weaning age, feed in availability and shortage. Goats are seasonal breeders; almost all the births are in September to October. The monthly weight measurements taken in dry season shows severe shortage of feed. In contrast to the result of this study, Zewdi et al. (2022) reported male kids and female kids having significantly different TMWT. The differences in TMWT observed between the sexes might be due to difference in hormones and physiological functions.

TMWT was highest in kids born in the wet season (8.03 ± 0.03 kg) and lower in dry season (7.68 ± 0.049 kg). Differences in TMWT in wet and dry season was that wet season could be associated with surplus and availability of browsing and grazing forages and in dry season the kids face prolonged and severe shortage of browsing forage and lack of crop residues. This study disagrees with the report of Dea and Eramo (2018); TMWT for Woyto Guji goats (9.32 ± 2.28 kg) is greater than that from the present study.

TMWT among possible pairs of years of birth had significant effect except between 2018 and 2022 and 2019 and 2021 which were non‐significant. The possible reasons for year‐wise variation in TMWT may be due to variation in the management and environmental conditions including feeding and uncontrolled factors of the year. Results obtained in the current study was nearly comparable with the same breed reported by Birhanie et al. (2018). However, TMWT of the Abergelle goat was smaller than Central Highland goat in the same management system (Abegaz et al. 2020) which is 9.32 ± 2.28 kg. On the other hand, under similar management system in CBBP on the same breed, the TMWT of the current study (7.5 ± 0.036 kg) is higher than TMWT (7.2 kg) reported by Amare et al. (2020).

TMWT in this study was 7.5 ± 0.03 kg, nearly comparable with the same breed reported (7.40 ± 0.09 kg) by Birhanie et al. (2018). But TMWT of the Abergelle goat was lighter compared with the Central Highland goat in traditional management system (Abegaz et al. 2020). Under a similar CBBP management system for the same breed, three‐month weight in the present study (7.5 ± 0.036 kg) was slightly higher than the 7.2 kg reported by Amare et al. (2020).

#### 3.1.3 SMWT

The overall LSM of SMWT was 8.8 ± 0.040 kg. Village and season had a highly significant effect on SMWT (*p* < 0.001) and year of birth had a significant effect (*p* < 0.05), while sex, parity and type of birth had no significant effect on SMWT, as shown in Table 3. The LSM ± SE of SMWT for the village (locations) was 9.8 ± 0.038 and 8.5 ± 0.090 kg in Addis Mender and Alquzu, respectively. The highest SMWT in Addis Mender village was due to the management of animal and availability of feed. Goat keepers in Addis Mender village use some improved forage crops (cowpea). In addition to this, there was mobility of animals to search surplus feed in shortage time by following the Tirari river around Zata, Korem and east Belsa (Wondim et al. 2021). The LSM of SMWT in the present study was 9.2 ± 0.05 kg for single kids and 9.18 ± 0.05 kg for twins, respectively.

**TABLE 3:** Least squares means (LSM ± SE) of average daily gain of Abergelle goats under up‐scaled CBBPs.

Source of variation	*N*	ADG1 (g/day) LSM ± SE	*N*	ADG2 (g/day) LSM ± SE	*N*	ADG3 (g/day) LSM ± SE	*N*	ADG4 (g/day) LSM ± SE
**Overall**	1335	58.2 ± 2.27	983	37.8 ±	937	19.2 ± 0.7	700	10.5 ± 0.12
**CV (%)**		25		45		57		66
**Sex of kids**		**		**		*		*
Male	671	64.6 ± 0.58^a^	493	48 ± 2.34^a^	439	29.2 ± 0.80^a^	439	19.2 ± 0.80^a^
Female	664	58.7 ± 0.58^b^	490	37.5 ± 2.3^b^	498	20.2 ± 0.75^b^	498	14.2 ± 0.75^b^
**Village**		***		**		***		**NS**
Addis Mender	275	64.9 ± 0.88^a^	268	48.86 ± 0.43^a^	230	40.460.78^a^	582	10.4 ± 0.64
Alquzu	1060	56.4 ± 0.449^b^	715	34 ± 0.71^b^	707	23 04 ± 0.45^b^	118	10.8 ± 0.29
**Birth type**		*		**NS**		*		**NS**
Single	1227	62.4 ± 0.42^a^	921	37.9 ± 0.57	892	29.9 ± 0.56^a^	670	10.6 ± 0.27
Twin	108	57.8 ± 1.44^b^	62	36.6 ± 2.2	45	16.1 ± 2.50^b^	30	9.1 ± 1.26
**Season**		*		NS		NS		*
Dry	431	55.01 ± 0.71^b^	334	38.5 ± 0.95	328	18.31 ± 0.92	286	9.36 ± 0.41^b^
Wet	904	59.7 ± 0.49^a^	649	37.5 ± 0.68	609	20.55 ± 0.68	414	11.30 ± 0.34^a^
**Year**		***		***		***		*
2018	319	60.89 ± 0.83^a^	215	20.2 ± 1.17^ab^	160	24.9 ± 1.31^a^	79	11.84 ± 0.78^a^
2019	208	54.75 ± 1.03^c^	161	16.8 ± 1.35^bc^	154	18.9 ± 1.33^bc^	136	9.19 ± 0.60^b^
2020	229	58.51 ± 0.98^abc^	164	17.6 ± 1.34^abc^	182	21.5 ± 1.22^ab^	114	10.3 ± 0.65^b^
2021	228	59.40 ± 0.98^ab^	173	22.0 ± 1.30^a^	160	19.2 ± 1.31^bc^	107	11.45 ± 0.67^a^
2022	351	56.78 ± 0.79^ac^	270	13.9 ± 1.04^c^	281	16.5 ± 0.9^c^	264	10.47 ± 0.43^b^
2023	208	54.75 ± 1.03^c^	161	16.8 ± 1.35^bc^	154	18.9 ± 1.33^bc^	136	9.19 ± 0.60^b^
**Parity**		***		*		*		*
1	239	57.69 ± 0.96^ab^	173	19.08 ± 1.31^c^	179	19.90 ± 1.2^bc^	137	10.70 ± 0.59^ab^
2	239	58.99 ± 0.96^ab^	178	18.70 ± 1.29^c^	168	19.85 ± 1.27^bc^	130	10.88 ± 0.61^ab^
3	230	63.54 ± 3.86a	181	33.69 ± 4.07^a^	183	43.18 ± 1.21^a^	117	9.54 ± 0.64^b^
4	242	55.5 ± 0.96^b^	188	17.29 ± 1.26^bc^	183	17.68 ± 1.58^c^	142	9.44 ± 0.58^b^
5	179	57.23 ± 1.11^ab^	131	14.84 ± 1.51^c^	109	16.72 ± 1.63^c^	99	12.37 ± 0.70^a^
6	191	59.09 ± 1.08^ab^	114	16.01 ± 1.61^bc^	102	17.77 ± 4.57^bc^	68	10.57 ± 0.84^ab^
> 7	15	60.33 ± 0.98^ab^	18	18.16 ± 1.28^b^	13	22.91 ± 4.57^b^	7	11.04 ± 2.64^ab^

*Note*: The least squares mean with different superscripts within the same column and class are statistically different.

Abbreviations: ADGs, average daily weight gain at different age groups; LSM, least squares means; SE, standard error.

NS, *p* > 0.05.

* *p* < 0.05.

** *p* < 0.01.

*** *p* < 0.001.

The single‐born kids were not significantly heavier in SMWT than twin‐born kids. This trend was not possibly a carryover effect of BWT and TMWT wherein single‐born kids had the highest respective weights. This might be because single‐born kids may not receive better nutrition or growth conditions compared to twin‐born kids during the specific measurement period for SMWT. And also sex had no significant effect on SMWT (*p* > 0.05). The present result disagrees with previous studies in other indigenous cross breeds (Belay et al. 2014; Teklebrha 2018) and agree with the same breed reported by Birhanie et al. (2018).

SMWT had substantial weight difference over the years. But SMWT did not linearly increase over the years. This stagnation could be attributed to several factors. One potential reason is the absence of precise selection of breeding bucks, implying that genetic factors influencing weight gain might not have been adequately addressed. Additionally, there may have been insufficient follow‐up procedures or gaps in the skills of data recorders, leading to inaccuracies in data collection. Furthermore, variations in the availability of feed and other environmental conditions from year to year could also contribute to the observed differences in goat weights. Seasonal fluctuations of weather condition had positive and negative effects on browsing shrubs and trees and sometimes can prolong dry season and increase shortage of feed. The current study of SMWT (8.8 ± 0.040 kg) of Abergelle goat disagree with the performance of Central Highland goats reported by Tesema et al. (2021) which is 10.6 ± 0.60 kg, and disagree with Woyto‐Guji goats under traditional management systems (13.32 ± 1.59 kg) reported by Dea and Eramo (2018). The result also disagrees with the performance of Bati, short‐eared Somali and Borana which is 16.31 ± 0.02, 13.9 ± 0.22 and 13.75 ± 0.36 kg, respectively (Gatew et al. 2019).

#### 3.1.4 NMWT

The overall LSM of NMWT was 12.1 ± 0.067 kg, as shown in Table 2. Sex, village, season and parity had a significant effect on NMWT (*p* < 0.05) but type of birth and year of birth had no significant effect on NMWT (*p* > 0.05), as shown in Table 2. The LSM ± SE of NMWT for the village was 14.1 ± 0.06 and 11.4 ± 0.12 kg in Addis Mender and Alquzu, respectively (*p* < 0.001). The LSM of NMWT for single and twin kids were 12.76 ± 0.11 and 12.69 ± 0.07 kg, respectively. This trend was possibly not a carryover effect of BWT and TMWT where single‐born kids had the highest respective weights. Both groups may have had access to similar quality and quantity of feed, allowing them to grow at the same rate and the genetic makeup of the goats could be similar enough that both single and twin kids exhibit comparable growth patterns. The NMWT in males and females had no significant effect (*p* > 0.05). The present result disagrees with previous studies on Sirohi goat type (15.29 ± 0.41 kg) (Waiz et al. 2018).

Year of birth was a significant source of variation for NMWT and pairwise differences were significant (2018–2019), but 2019–2020 and 2018–2023 had no significant effect. NMWT across year of birth showed that highest NMWT was recorded in 2020 and lowest NMWT in 2021, 2020 and 2018. This may be associated with the lack of accurate buck selection, inadequate follow‐up and gaps in enumerator's data recording skill. The other reason could be the difference of year‐to‐year variation in the availability of feed and other environmental conditions. NMWT of Abergelle goat in scale‐up CBBP village was 12.1 ± 0.067 kg not comparable with NMWT of Central Highland goat (17.76 ± 1.01 kg) reported by Abegaz et al. (2020) and in line with the same breed (Amare et al. 2020) which is 12.76 ± 0.26 kg under CBBP areas. However, under traditional management system the present study on NMWT was lower than Woyto‐Guji goats, which is 15.89 ± 2.94 kg (Dea and Eramo 2018). NMWT in year had trends of decreasing except from 2019 to 2020; this might be the inefficient use of breeding bucks and the premature sale of breeding bucks in CBBP of Abergelle goat (Mueller et al. 2021).

The implication of these results is that factors such as sex, village, season and parity significantly influence the NMWT of Abergelle goats, while the type of birth and years of birth do not have a significant impact on NMWT. Year of birth also plays a crucial role in NMWT as source variation, with certain years showing significant differences in NMWT. This study suggests the importance of considering these factors in breeding and management practices to optimize the weight of Abergelle goats in various contexts, such as CBBP or traditional management systems.

#### 3.1.5 YWT

In the present study, the overall LSM ± SE of YWT was 15.3 ± 0.04 kg. The effects of birth type, parity and sex were non‐significant on YWT (*p* > 0.05), as shown in Table 3. This study revealed that multiple birth was encouraged because at market age the single‐ and twin‐born animals had similar weight. The LSM ± SE of YWT were 16.1 ± 0.17 and 14.9 ± 0.10 kg in Addis Mender and Alquzu, respectively. The pair‐wise comparison of the means showed that YWT differences between pairs of village was highly significant (*p* < 0.001). Single‐ and twin‐born animals had YWT of 15.43 ± 0.14 and 15.37 ± 0.15 kg, respectively, that is, birth type had no significant effect on YWT. This effect may be attributed to lesser availability of uterine space (horns) within twin birth affecting prenatal nutrition/development and also competition for dams' milk during pre‐weaning period but had no significant effect on YWT (*p* > 0.05). The present study disagrees with the same breed documented by Amare et al. (2020), which is 17.00 ± 0.77 and 16.13 ± 0.24 kg for single‐ and twin‐born animals, respectively. Other authors mentioned that the differences in YWT between the sexes might be due to difference in hormones and physiological functions between the two sexes (Tesema et al. 2021; Deribe and Taye 2014). YWT was lower in dry season (14.6 ± 0.16 kg) and higher in wet season (15.8 ± 0.103 kg); wet season possibly provided optimum conditions for growth of animals. The variation in kid's growth up to yearling age in the dry season was distinct from those observed in the wet season. The seasonal impact correlates with variations in both feed availability and disease prevalence (Farrag 2022). The YWT differed significantly among potential pairs of birth years, notably between 2018 and 2019, 2018 and 2021 and 2018 and 2023, as well as between 2019 and subsequent years. However, there was no significant effect on YWT observed between 2019 and 2021 and 2020 and 2023.

#### 3.1.6 Daily Weight Gain of Growth Traits

Average daily gain (ADG) serves as a fundamental measure for evaluating growth rates across various animal species. A quicker rate of growth enabled animals to reach market weights in a shorter timeframe while utilizing minimal resources. Table 3 presents the LSM and respective standard errors for the analysis of growth rates across different age groups. Notably, the ADG of young animals exhibited a declining trend as their age increased. Specifically, the weight gained from birth to the weaning stage is only half of the weight gained post‐weaning. This result aligns with previous studies by Tesema et al. (2021) and Deribe and Taye (2023b). This outcome is not unexpected, as the pre‐weaning weight gain of young animals is closely linked to factors such as the nutritional status of the mother, maternal care and the dam's milk production capacity. Conversely, the post‐weaning growth of young animals is predominantly influenced by their genetic predisposition and other environmental factors. The effect of parity, village, birth types and year of birth, sex and season on average daily weight gain and LSM of these traits are presented in Table 3. This result was unsurprising due to the close correlation between pre‐weaning weight gain in young animals and factors such as the nutritional status of the dam, mothering capability and milk production potential of dam, in addition to genetic predisposition and environmental influences. However, post‐weaning growth in young animals is primarily determined by their genetic potential and other environmental factors (Tesema et al. 2020).

#### 3.1.7 Daily Weight Gain From Birth to 3 Months of Age (ADG1)

The overall ADG1 (in grams) from birth to 3 months of age was 58.2 ± 2.27 g/day. The effects of village, season, birth type, year and parity on ADG1 were significant (*p* < 0.01), whereas the effects of sex was non‐significant (Table 4). The LSM ± SE of ADG1 of the village were 64.9 ± 0.88 and 56.4 ± 0.449 g/day in Addis Mender and Alquzu, respectively. The differences between villages was highly significant (*p* < 0.01) and mean ADG1 for single kids and twins were 62.4 ± 0.42 and 57.8 ± 1.44 g/day, respectively. The ADG1 means of single kids differed significantly from twin kids. Sex had no significant effect on ADG1; male and female kids had similar ADG from birth to 3 months of age. The LSM of ADG1 across the year showed that all pair‐wise comparisons of means were significant (2018–2019, 2018–2020, 2021–2023, 2018–2023) (*p* < 0.05) but pairs 2019 and 2020 were non‐significant. Gatew et al. (2019) reported higher which is 86.22 ± 2.02 g/day for Bati goat ADG1 in traditional management system compared with the current result. This difference might be attributed to both genetics (as Abergelle goat is small in size) and also poor management. The current result was comparable with Central Highland breed reported by Tesema et al. (2021) which is 54.1 ± 4.11 g/day. The ADG1 of this study was less than Woyto Guji goat breed reported by Dea and Eramo (2018) that was 62.32 ± 24.67. Similarly, the current result (58.2 ± 2.27 g/day) is less than the result of Deribe and Taye (2013b), which is 76.6 ± 2.3 g/day for Abergelle breed under semi‐intensive management system. This may be because the sample size number of animals in the experiment was smaller than the current study, feed supplementation and the dynamic environmental factors. When compared with Abergelle crossed with Bore goat first generation the current result was less than that reported by Belay et al. (2014) which is 73 ± 113 g/day, and lower than the values reported by Zergaw et al. (2016) who reported 73.93 ± 2.01 for Woyto Guji goat.

**TABLE 4:** Estimates of (co)variance components and genetic parameters for body weights in Abergelle goat.

Trait	Model	*σ* ^2^ *_a_*	*σ* ^2^ *_m_*	*σ_am_*	*σ* ^2^ *_c_*	*σ*2*_e_*	*σ* ^2^ *p*	*h* ^2^ *_a_* ± SE	*h* ^2^ *_m_* ± SE	*r_am_*	*c* ^2^ ± SE	*e* ^2^ ± SE	*h* ^2^t	LogL
BWT	1	0.04	—	—		0.05	0.09	0.40 ± 0.05				0.57 ± 0.05	0.44	1579.8
	2	0.03	0.01			0.06	1.00	0.23 ± 0.06	0.01 ± 0.03			0.52 ± 0.05	0.03	1574.9
	**3**	**0.04**	**0.03**	**−0.01**		**0.04**	**0.08**	**0.34** ± **0.070**	**0.11** ± **0.07**	**−0.72**		**0.48** ± **0.08**	**0.50**	**1584.6**
	4	0.01			0.00	0.04	1.00	0.20 ± 0.06			0.01 ± 0.03	0.79 ± 0.05	0.01	1578.3
TMWT	1	0.54				0.77	1.31	0.33 ± 0.05				0.67 ± 0.05	0.41	−1242.2
	2	0.37	0.11			0.84	1.32	0.28 ± 0.05	0.08 ± 0.03			0.64 ± 0.05	0.28	−1237.9
	3	1.02	0.89	−0.72		0.93	2.84	0.34 ± 0.11	0.27 ± 0.07	−0.84		0.41 ± 0.08	0.36	−1238.1
	**4**	**0.28**			**0.11**	**0.82**	**1.10**	**0.45** ± **0.062**			**0.08** ± **0.03**	**0.62** ± **0.05**	**0.25**	−**1237.7**
SMWT	1	0.77				0.88	1.65	0.5 ± 0.05				0.51 ± 0.05	0.47	−1276.3
	2	0.72	0.18			0.79	1.69	0.39 ± 0.07	0.10 ± 0.04			0.51 ± 0.05	0.43	−1266.4
	**3**	**1.34**	**0.66**	−**0.55**		**0.77**	**2.77**	**0.40** ± **0.040**	**0.22** ± **0.08**	−**0.71**		**0.30** ± **0.08**	**0.48**	−**1257.2**
	4	0.67			0.018	0.75	1.42	0.44 ± 0.06			0.10 ± 0.03	0.48 ± 0.05	0.47	−1266.3
NMWT	1	0.82				1.90	2.72	0.32 ± 0.06				0.69 ± 0.06	0.30	−1343.4
	**2**	**0.7**	**0.16**			**1.82**	**2.68**	**0.42** ± **0.07**	**0.04** ± **0.04**			**0.68** ± **0.06**	**0.26**	−**1337.1**
	3	0.86	0.05	−0.05		2.45	2.68	0.30 ± 0.14	0.02 ± 0.09	−0.78		0.45 ± 0.10	0.32	−1338.7
	4	0.71			0.010	1.70	2.68	0.26 ± 0.07			0.04 ± 0.04	0.68 ± 0.06	0.26	−1337.1
YWT	1	2.03				3.88	5.91	0.46 ± 0.06				0.56 ± 0.06	0.34	−1655.6
	**2**	**2.54**	**0.67**			**3.43**	**6.64**	**0.41** ± **0.14**	**0.08** ± **0.05**			**0.54** ± **0.06**	**0.38**	−**1648.6**
	3	2.38	0.82	−0.20		3.11	6.11	0.36 ± 0.13	0.11 ± 0.10	−0.83		0.50 ± 0.09	0.39	−1648.6
	4	2.75			0.054	3.90	7.19	0.42 ± 0.07			0.07 ± 0.04	0.53 ± 0.07	0.38	−1648.9

Abbreviations: *σ* ^2^ *a*, direct additive variance; *σ* ^2^ *c*, maternal permanent environmental variance; *σ* ^2^ *e*, residual variance; *σ* ^2^ *m*, maternal additive variance; *σ* ^2^ *p*, phenotypic variance; *σam*, direct‐maternal genetic covariance; BWT, birth weight; *c* ^2^, maternal permanent environmental variance as a proportion of phenotypic variance; *h* ^2^ *a*, the heritability of direct additive effects; *h* ^2^ *m*, heritability of maternal additive effects; *h* ^2^t, total heritability; NMWT, 9‐month weight; *ram*, the correlation between direct and maternal genetic effects; SMWT, 6‐month weight; TMWT, 3‐month weight; YWT, yearling weight.

The ADG of kids showed decreasing trend as the age increased, that is, the weight gain from birth to weaning age was one fold of the post‐weaning weight gain of kids. This result concurs (Gatew et al. 2019) with Hulunim and Deribe and Taye (2023b). This was because, in addition to genetic potential and other environmental factors, pre‐weaning weight gain of kids are closely associated with the nutritional status of dam, mothering ability and milk production potential of the dam. However, the growth of kids after weaning is largely dependent on their genetic potential and levels of other environmental influences.

#### 3.1.8 Daily Weight Gain From 3 to 6 Months of Age (ADG2)

The overall ADG2 (in grams) from 3 to 6 months of age was 37.8 ± 3.62 g/day, as shown in Table 3. The effects of village, sex, and parity on daily gain from 3 to 6 months of age were highly significant (*p* < 0.01), whereas the effects of year, season and type of birth were non‐significant. Weight gain from 3 to 6 months of age was higher in Abergelle goat than Woyto Guji goat reported by Zergaw et al. (2016) which is 31.92 ± 2.33 and in the current study is also smaller than Central Highland goat by the same author which is 65.4 ± 2.84 kg (Zergaw et al. 2016). The LSM ± SE of ADG2 of scale‐up village were 48.86 ± 0.43^a^ and 34 ± 0.71^b^ g/day in Addis Mender and Alquzu village, respectively. The current study results showed that CBBP scale‐up village for ADG2 were significantly different from one another. The LSM of ADG2 for type of births was non‐significant, twins (36.6 ± 2.2 g/day) and single (37.9 ± 0.57 g/day) (*p* > 0.05). The non‐significant difference of ADG2 twin‐ and single‐birth kids observed in the present study is in line with the trends shown in Table 3 with BWT and contradicts the trend of TMWT, as shown in Table 3. The possible reason may be that postnatal competition was eliminated after 3 months of age and thus twins expressed their potential in ADG2. The current result was lower than the previous reports on Central Highland goat which is 71.07 ± 5.61 and 63.61 ± 5.06 g/day and higher than Woyto‐Guji which is 31.93 ± 2.59 and 30.31 ± 2.7 g/day reported by Zergaw et al. (2016). Male kids had higher average ADG2 than females (48 ± 2.34 vs. 37.5 ± 2.3 g/day), respectively.

#### 3.1.9 Daily Weight Gain From 6 to 9 Months of Age (ADG3)

The overall ADG3 from 6 to 9 months of age (in grams) was 19.2 ± 0.7 g/day. The effects of CBBP scale‐up village, parity, year and season on ADG3, as shown in Table 3, were significant (*p* < 0.05), whereas the effects of birth type and sex were non‐significant (Table 3). Village had highly a significant effect on ADG3 (*p* < 0.01); in Addis Mender village, relatively, the browsing shrubs and natural trees as the animal feed are in surplus than in Alquzu village. Management of animals and searching of feed for their animals at shortage of feed was the best in Addis Mender (Wondim et al. 2021).

#### 3.1.10 Daily Weight Gain From 9 Months up to Yearling Age (ADG4)

The overall ADG4 (in grams) from 9 months age up to yearling age was 10.5 ± 0.27 g/day. The effects of season, sex, year and parity on ADG4 were significant (*p* < 0.05), whereas the effects of village and birth type was non‐significant (*p* > 0.05), as shown in the Table 3. The LSM ± SE of ADG4 of the village were 10.4 ± 0.64 and 10.8 ± 0.29 g/day in Addis Mender and Alquzu, respectively. The differences between village was non‐significant (*p* > 0.05) and LSM of ADG4 for single kids and twins were 10.6 ± 0.27 and 9.1 ± 1.26 g/day, respectively. And the sex of kids on daily weight gain was significant at *p* < 0.05, the maximum daily gain and significant changes obtained from birth up to 6 months of age. The LSM of ADG4 years showed that all pair‐wise comparisons of means were not significant.

## 4 Genetic Parameters Estimation for Abergelle Goats in CBBP Scale‐Up Village

### 4.1 Heritability Estimates for Growth Traits

Genetic parameters are very important tools that help to give decision on the fate of individuals and on the strategy of how to exploit the population in a sustainable manner. It includes heritability (*h* ^2^), repeatability (*r*) and correlation estimates of direct heritability for growth traits in goats in CBBP scale‐up villages, as shown in Table 4. Heritability results at different age groups for growth trait ranged from low to high. The estimates of direct heritability (*h* ^2^) for BWT, TMWT, SMWT, NMWT and YWT were 0.34 ± 0.070, 0.45 ± 0.062, 0.40 ± 0.040, 0.42 ± 0.07 and 0.41 ± 0.14 respectively. Direct heritability in BWT was low from other growth traits. Direct heritability (*h* ^2^) reflects the proportion of phenotypic variance attributable to direct genetic effects. High direct heritability estimate (*h* ^2^) was observed for TMWT (0.45 ± 0.062) and potentially high influence of genes on the body weight of kids at 3 months of age. This study agrees on TMWT direct heritability on Nigerian Sahalian goat and similar to small ruminant on Bharat Merino sheep that direct heritability was high for TMWT (Otuma and Osakwe 2014; Mallick et al. 2021). Notably, the direct heritability demonstrates increase across successive growth stages, starting from BWT to TMWT then decrease to SMWT. The present result is in line with the report of Gowane et al. (2015) and Tesema et al. (2020), who observed a decline up to 6 months followed by an increase up to the yearling age and is also similar to the report for Arsi‐Bale goats (Mohammed et al. 2013). In the present study, direct heritability of TMWT was greater than Balkan goat which is 0.110 ± 0.044 (Caro‐Petrović et al. 2012). However, the relative contribution of direct additive genetic effect for each growth trait can vary depending on the management practices of animals (Shenkut et al. 2022). In the present study area, the environmental factors might significantly influence the expression of genes related to the growth traits in goats. Management practice such as feeding, housing conditions, sanitation and handling procedures can influence goat behaviour, stress levels and susceptibility to diseases. The variation of these management practices can affect the genetic parameters estimates, especially if not all these factors are considered in the model.

The harsh climate and weather condition environmental variables like temperature, humidity and precipitation can affect goat productivity and health. Extreme weather events or seasonal changes may impact traits such as milk production, growth rates and disease resistance, complicating genetic evaluations. Goats are susceptible to external parasites, including worms, ticks and lice. Parasite infestations can reduce feed efficiency, stunt growth and weaken immune responses, potentially masking genetic differences among animals. Health status and disease outbreaks or chronic health issues within a goat population can influence the accuracy of genetic parameter estimates. The estimate of direct heritability in SMWT in this study exceeds those reported by Rout et al. (2018) for the Jamunapari goat breed (0.14 ± 0.04), Markhoz goats (0.22 ± 0.05), (Baneh et al. 2012) Naeini goats (0.25 ± 0.05) and (Latifi and Razmakar 2019) Markhoz goat breed (0.25 ± 0.05). However, it is in line with the values reported by Shaat and Mäki‐Tanila (2009) for Nigerian Sahelian goats (0.41 ± 0.08), (Mohammed et al. 2013) for Arsi‐Bale goats (0.39 ± 0.08), (Shivanand et al. 2022) for Sirohi goats (0.39 ± 0.05) and similar for Egyptian Zaraibi goats (0.43 ± 0.05) as reported by Waiz et al. (2018). The direct heritability (*h* ^2^) NMWT was determined to be 0.42 ± 0.07 in the scale‐up CBBP village. The differences in the estimates among these investigations can be attributed to variation in data quantity, recording accuracy, management techniques, utilized methodologies and potential impacts from inbreeding (in this study the inbreeding coefficient is zero and the breeding bucks are allocated randomly). Direct heritability of YWT of Abergelle in the present study was 0.41 ± 0.14 which agree with the report of Otuma and Osakwe (2014). In general, weight traits heritability range from low to high. This result had the implication that high estimated heritability indicates that lives of the animals might be simply improved by selection of breeding bucks.

Selection of appropriate model is vital to increase the accuracy of estimates and may affect the decisions of the breeder. The best models are highlighted in bold. Analysis of four different models, based on the logL ratio test, shows that the most suitable models for BWT, TMWT, SMWT, NMWT and YWT, as shown in Table 4, are Models 3, 4, 3, 2 and 2, respectively.

#### 4.1.1 Phenotypic and Genetic Correlations

The estimates of phenotypic and genetic correlations for different growth traits obtained from the bivariate analysis of animal model are presented in Table 5. Phenotypic correlations include both genetic and environmental variation while genetic correlations merely evaluate genetic contributions obtained from common ancestral relationships. Genetic correlations among growth traits in this study were higher than the corresponding phenotypic correlation values. This agreed with the result reported by Tesema et al. (2020). Phenotypic correlations of BWT with later growth stages were low in comparison to the reports of Tesema et al. (2020) and Mohammed et al. (2013). On the other hand, correlation values for TMWT–SMWT, SMWT–NMWT and NMWT–YWT are higher and positive which are comparable with the results of Mohammed et al. (2013) and Tesema et al. (2020) for indigenous Arsi‐Bale and Central Highland × Boer goat breeds, respectively. These higher phenotypic correlation values mean that kids at 3 months of age with heavier weight tend to attain heavier weight at later stages of growth.

**TABLE 5:** Phenotypic (below diagonal) and genetic (above diagonal) correlation for growth traits.

	BWT	TMWT	SMWT	NMWT	YWT
BWT	**1.00**	0.23 ± 0.014	0.12 ± 0.128	0.09 ± 0.15	0.091 ± 0.04
TMWT	0.068 ± 0.02	**1.00**	0.87 ± 0.043	0.67 ± 0.071	0.71 ± 0.017
SMWT	0.044 ± 0.025	0.84 ± 0.08	**1.00**	0.89 ± 0.053	0.72 ± 0.05
NMWT	0.029 ± 0.026	0.65 ± 0.02	0.68 ± 0.017	**1.00**	0.74 ± 0.014
YWT	0.018 ± 0.043	0.42 ± 0.03	0.47 ± 0.026	0.58 ± 0.03	**1.00**

Abbreviations: BWT, birth weight; NMWT, 9‐month weight; SMWT, 6‐month weight; TMWT, 3‐month weight; YWT, yearling weight.

Phenotypic correlation is a statistical measure that evaluates the strength and direction of the relationship between two observable traits. In the context of this study, the focus is on the BWT of Abergelle goats and its association with other growth traits. When examining BWT in relation to other traits such as TMWT, SMWT, NMWT and YWT, the genetic correlation coefficients, which quantify the strength of these associations, ranged from 0.091 to 0.232. Despite being moderate, these correlations were not particularly strong, suggesting that while there is some relationship between BWT and the other traits, it may not be significant enough to base decisions solely on BWT when considering the growth of the animal. Additionally, higher correlations were found among the other growth traits (SMWT, NMWT and YWT), indicating when one of these traits increases, the others tend to increase as well.

Moving on to genetic correlation, this measure assesses the extent to which the variation in one trait is attributable to genetic factors that also influence another trait. In the case of BWT and other growth traits, the genetic correlations were generally small, indicating weak genetic relationships with later growth stages. However, there was a high genetic correlation between TMWT and other traits (SMWT, NMWT and YWT), ranging from 0.71 to 0.87. This suggests that while there may be some genetic influence on the relationship between BWT and TMWT, it is not particularly strong. Conversely, there were higher positive genetic correlations observed among the later growth stages (TMWT–SMWT, SMWT–NMWT, NMWT–YWT and SMWT–YWT), with correlation coefficients ranging from 0.74 to 0.89. This indicates that genetic factors influencing one growth trait tend to also influence the others positively, highlighting the concept of pleiotropy where one gene affects multiple traits.

The result of this study indicates that early selection based solely on BWT may not be effective in breeding programmes aimed at improving the growth of Abergelle goats. Instead, considering the genetic correlations among TMWT and other growth traits indicates the possibility of indirect selection for SMWT, NMWT and YWT at early age using TMWT. In general, recognizing and understanding genetic correlations among growth traits is crucial for optimizing breeding schemes and ultimately improving the productivity of goat populations. This knowledge can aid breeders in making informed decisions that lead to more effective selection and breeding outcomes.

#### 4.1.2 Genetic Parameter Estimation and Heritability for Average Daily Body Weight Gain at Different Age Categories

The estimates of (co)variance components and genetic parameters for ADG of Abergelle goats, as shown in Table 6, shows that the magnitude of genetic and environmental effects varied across growth stages and according to the animal model fitted. Across growth rate traits, direct additive heritability ranged from low to moderate (0.01–0.31), indicating that genetic improvement through selection is feasible, although the expected response to selection differs with age. The highest direct heritability was observed for ADG3 under Model 1 (0.31 ± 0.07) and ADG1 under Model 3 (0.27 ± 0.05), whereas the lowest estimate was recorded for ADG2 under Model 3 (0.01 ± 0.00). These results suggest that additive genetic effects contribute more substantially to growth before weaning and during the post‐weaning growth phase from 6 to 9 months than during the early post‐weaning period. Similar age‐dependent changes in heritability for growth traits have been reported in indigenous and commercial goat breeds, where heritability generally increases with age as maternal influences decline and the animal's own genotype becomes more fully expressed (Yiga‐Kibuuka et al. 2024; Mokhtari et al. 2024; Singh et al. 2022) Maternal genetic effects were particularly important for ADG1 and ADG3, with maternal heritability estimates reaching 0.24 under Model 3. This result highlights the significant contribution of dam performance, particularly maternal ability and milk production, to early growth of kids. The inclusion of maternal genetic effects substantially reduced estimates of direct heritability in Models 2 and 3 compared with Model 1, demonstrating that exclusion of maternal effects may increase estimates of additive genetic variance. Similar observations have been reported for Boer, Angora, Black Bengal and indigenous African goats, where fitting maternal genetic effects resulted in more accurate partitioning of phenotypic variance and improved estimation of breeding values (Jalil et al. 2020; Visser and Snyman 2023). The direct‐maternal genetic correlations estimated in Model 3 were consistently high and positive (0.86–0.96). These results indicate that genes influencing direct growth also tend to improve maternal performance in Abergelle goats. Positive direct‐maternal correlations have been reported in several goat populations and suggest that simultaneous improvement of kid growth and maternal ability is possible through selection (Visser and Snyman 2023). However, the very high estimates observed in the present study should be interpreted cautiously because estimates of direct‐maternal covariance are often associated with relatively large sampling errors, limited pedigree depth and strong dependence on the fitted statistical model.

**TABLE 6:** Estimates of (co)variance components and genetic parameters for weight gain of Abergelle goats.

Trait	Model	*σ* ^2^ *_a_*	*σ* ^2^ *_m_*	*σ_am_*	*σ* ^2^ *_c_*	*σ*2*_e_*	*σ* ^2^ *p*	*h* ^2^ *_a_* ± SE	*h* ^2^ *_m_* ± SE	*r_am_*	*c* ^2^ ± SE	*e* ^2^ ± SE	*h* ^2^t	LogL
ADG1	1	260.6				702.4	974.7	0.26 ± 0.09				0.72	0.27	−2353.6
	**2**	**170.0**	**98.2**			**700.4**	**951.7**	**0.18** ± **0.04**	**0.1** ± **0.03**			**0.74** ± **0.5**	**0.23**	−**2345.6**
	3	398	356.5	352.6		406.1	1500	0.27 ± 0.05	0.24 ± 0.07	0.94		0.27 ± 0.08	0.74	−2356.4
	4	483.1			126	438.2	2340	0.2 ± 0.04			0.05 ± 0.002	0.19 ± 0.007	0.21	−2345.8
ADG2	1	64				304.2	374.6	0.17 ± **0.05**				0.81 ± **0.07**	0.17	−1678.4
	2	67	45			301.3	379.5	0.18 ± 0.07	0.12 ± 0.05			0.79 ± 0.05	0.24	−1670.8
	3	2.8	84.5	14.9		280.4	374.2	0.01 ± **0.00**	0.23 ± **0.05**	0.96		0.75 ± **0.08**	0.18	−1657.5
	**4**	**12.4**			**36.4**	**293.2**	**375.4**	**0.03** ± **0.001**			**0.10** ± **0.02**	**0.78** ± **0.4**	**0.03**	−**1652.22**
ADG3	1	158.4				368.6	506.8	0.31 ± **0.07**				0.73 ± **0.5**	0.31	−1409.26
	2	48.9	43.6			368.4	210.3	0.11 ± **0.02**	0.09 ± **0.001**			0.80 ± **0.04**	0.2	−1450.56
	3	136.4	122.8	122		433.0	519.4	0.26 ± **0.08**	0.24 ± **0.04**	0.94 ± **0.7**		0.83 ± **0.5**	0.073	−1401.45
	**4**	**94.6**			**73.8**	**341.6**	**510.0**	**0.19** ± **0.04**			**0.14** ± **0.02**	**0.67** ± **0.4**	**0.19**	−**1401.19**
ADG4	1	98.1				499.3	617.4	0.16 ± 0.04				0.81 ± 0.4	0.16	−1101.59
	2	60.2	42.1			485.7	616.4	0.10 ± 0.001	0.07 ± 0.003			0.79 ± 0.4	0.13	−1101.29
	**3**	**125.1**	**98.5**	**95.3**		**541.2**	**662.9**	**0.19** ± **0.04**	**0.15** ± **0.006**	**0.86** ± **07**		**0.82** ± **0.5**	**0.48**	−**1100.99**
	4	58.9			100.4	417.8	621.5	0.09 ± 0.007			0.16 ± 0.02	0.67 ± 0.04	0.09	−1199.98

Abbreviations: *σ* ^2^ *a*, direct additive variance; *σ* ^2^ *m*, maternal additive variance; *σ* ^2^ *c*, maternal permanent environmental variance; *σ* ^2^ *e*, residual variance; *σ* ^2^ *p*, phenotypic variance; *σam*, direct‐maternal genetic covariance; ADG1, average daily gain from birth to weaning age; ADG2, average daily gain from weaning to 6 months; ADG3, average daily gain from 6 to 9 months; ADG4, average daily weight gain from 9 months to yearling weight; *c* ^2^, maternal permanent environmental variance as a proportion of phenotypic variance; *h* ^2^ *a*, the heritability of direct additive effects; *h* ^2^ *m*, heritability of maternal additive effects; *h* ^2^t, total heritability; *ram*, the correlation between direct and maternal genetic effects.

Maternal permanent environmental effects accounted for 5%–16% of the phenotypic variance, with the largest contribution observed for ADG4. These estimates indicate that non‐genetic maternal influences, including uterine environment, milk yield, maternal behaviour, nutrition and management during early life, continued to affect offspring growth beyond weaning. Similar maternal permanent environmental effects have been reported in tropical goat production systems, emphasizing the importance of improving doe nutrition and management alongside genetic selection (Atoui et al. 2022; El‐Raghi and Hashem 2022). Residual variance represented the largest proportion of the phenotypic variance across all ADG traits (0.67–0.83), indicating that environmental factors remain the major source of variation in growth rate. Feed availability, seasonal fluctuations, disease challenge, grazing conditions and management practices are well‐known determinants of growth performance under extensive and semi‐intensive production systems typical of Ethiopian indigenous goats. Consequently, improvements in nutrition, health management and husbandry practices are expected to complement genetic improvement and substantially increase growth performance (Oloo et al. 2024). Comparison of logL values indicates that models accounting for maternal genetic or maternal permanent environmental effects generally provided a better fit than the simple direct additive model, particularly for ADG2 and ADG4. This finding confirms that maternal effects constitute an important source of variation for growth traits in Abergelle goats and should be considered during genetic evaluation. Overall, the moderate estimates of direct heritability for ADG1 and ADG3 demonstrate sufficient additive genetic variation to support selection for improved growth rate. However, because maternal genetic and permanent environmental effects contributed appreciably to early growth, genetic evaluation programmes for the Abergelle goat CBBP should employ animal models that appropriately account for maternal influences to obtain unbiased breeding values and maximize long‐term genetic gain.

## 5 Conclusion

The CBBP for Abergelle goats showed encouraging genetic improvement in growth traits. Both environmental and genetic factors significantly influenced goat performance, with moderate to high heritability estimates indicating good potential for further selection and breeding progress. Positive correlations among growth traits also suggest that improving one trait can positively affect others, especially when selection is done at an early age.

Although the genetic trend was promising, the rate of progress remains inconsistent and lower compared to other goat breeds. Therefore, the breeding programme should be optimized through better sire selection, increased selection intensity, accurate data recording, effective sire rotation and selecting bucks at 6 months of age to retain genetically superior animals within the community.

## Author Contributions

**Yeshiwas Walle Abebe**: conceptualization (lead), data collection entry, management and analysis, writing – original draft (lead), formal analysis (lead), writing – review and editing, methodology. **Wossenie Shibabaw Meberatie**: conceptualization (supporting), methodology, first draft writing (supporting), supervision, validation of the methodology and analysis. **Zeleke Tesema Wondie**: conceptualization (supporting), methodology, first draft writing (supporting), supervision, validation of the methodology and analysis.

## Funding

The authors have nothing to report.

## Ethics Statement

Before any attempt, the protocol was approved by chairman Abebe Tibebu Fikadu of Amhara Agricultural Research Institute and Sekota Dryland Agricultural Research Center Ethical Committee (approval number: SDARC/12/03/06/2018 (Annex 8)). Official permission was also obtained from livestock and fishery offices of the study area as well as goat farm owners. Necessary changes and revision were carried out as per the feedback from the committee board before moving ahead to the data collection scheme.

## Conflicts of Interest

The authors declare no conflicts of interest.

## Supporting information




**Supporting File**: vms371202‐sup‐0001‐SuppMat.docx

## Data Availability

The data used in this study are available from the corresponding author upon reasonable request.
