# Supplementary material for: The Estimation of Phenotypic and Genetic Parameters for Growth Rate Traits of Abergelle Goat Under the Community‐Based Breeding Programme in the North‐Eastern Parts of Ethiopia
Source: Vet Med Sci. 2026 Aug 29;12(5):e71202. doi: 10.1002/vms3.71202 (PMC13525851; doi:10.1002/vms3.71202)
Supplement: Supplementary file 1 [file VMS3-12-e71202-s001.docx]

Codes used to run wombat software

A single optional comment line of up to 74 characters

COMMENT WOMBAT analysis of Abergelle goat birth weight

\# The type of analysis to be performed. In this case a UNIvariate #analysis

ANALYSIS UNI

\# Name the pedigree file, assuming it is in the same folder as #the parameter file

PEDS pedfile.txt

\# Name the data file, assuming it is in the same folder as the #parameter file

DATA datafile.txt

\# Description of the data file

\# Each variable to be fitted in the model needs to be followed by

\# the maximum number of levels

ANIMAL 1989

sire	54

MOTHER 635

SEX 2

PARITY 7

PPWT

BWT

END DATA

\# Model specification

\# Type of effect (FIXed, COVariate, RANdom) and variable name

\# NRM indicates that a pedigree is available for ANIMAL

MODEL

FIX SEX

RAN ANIMAL NRM

\# The trait to be analysed

TRAIT BWT

END

\# Specify starting values and the number of rows and columns in #the matrix

\# These are not very important here

VAR residual 1

0.1

VAR ANIMAL 1

0.1

A single optional comment line of up to 74 characters

COMMENT WOMBAT analysis of Abergelle goat six month weight

\# The type of analysis to be performed. In this case a UNIvariate analysis

ANALYSIS UNI

\# Name the pedigree file, assuming it is in the same folder as the parameter file

PEDS pedfile.txt

\# Name the data file, assuming it is in the same folder as the parameter file

DATA datafile.txt

\# Description of the data file

\# Each variable to be fitted in the model needs to be followed by

\# the maximum number of levels

ANIMAL 1211

sire	54

MOTHER 428

SEX 2

PARITY 7

PPWT

SMWT

END DATA

\# Model specification

\# Type of effect (FIXed, COVariate, RANdom) and variable name

\# NRM indicates that a pedigree is available for ANIMAL

MODEL

FIX SEX

RAN ANIMAL NRM

\# The trait to be analysed

TRAIT SMWT

END

\# Specify starting values and the number of rows and columns in the matrix

\# These are not very important here

VAR residual 1

1.0

VAR ANIMAL 1

1.0
